# Thy-1 (CD90) Signaling Preferentially Promotes RORγt Expression and a Th17 Response

**DOI:** 10.3389/fcell.2018.00158

**Published:** 2018-11-23

**Authors:** Suzanne Furlong, Melanie R. Power Coombs, Javad Ghassemi-Rad, David W. Hoskin

**Affiliations:** ^1^Department of Microbiology and Immunology, Dalhousie University, Halifax, NS, Canada; ^2^Department of Pathology, Dalhousie University, Halifax, NS, Canada; ^3^Department of Surgery, Dalhousie University, Halifax, NS, Canada

**Keywords:** CD90, cytokine synthesis, glycosylphosphatidylinositol-anchored protein, T cell, Thy-1

## Abstract

Thy-1 (CD90) is a glycosylphosphatidylinositol-anchored protein (GPI-AP) with signaling properties that is abundant on mouse T cells. Upon antibody-mediated crosslinking, Thy-1 provides a T cell receptor (TcR)-like signal that is sufficient to drive CD4^+^ T cell proliferation and differentiation into effector cells when costimulatory signals are provided by syngeneic lipopolysaccharide-matured bone marrow-derived dendritic cells. In this study, we investigated the impact of Thy-1 signaling on the production of the T helper (Th) cell subset-associated cytokines, interferon (IFN) γ, interleukin (IL)-4 and IL-17A, as well as the *in vitro* polarization of highly purified resting CD4^+^ T cells into Th1, Th2, and Th17 cells. Although CD8^+^ T cells expressed more Thy-1 than CD4^+^ T cells, both T cell populations were equally responsive to Thy-1 stimulation. In contrast to TcR stimulation of CD3^+^ T cells, which favored IFNγ and IL-4 production, Thy-1 signaling favored IL-17 synthesis, indicating a previously unidentified difference between the consequences of Thy-1 and TcR signal transduction. Moreover, Thy-1 signaling preferentially induced the Th17-associated transcription factor RORγt in CD4^+^ T cells. As with TcR signaling, Thy-1 stimulation of CD4^+^ T cells under the appropriate polarizing conditions resulted in Th1, Th2 or Th17 cell induction; however, Thy-1 stimulation induced nearly 7- and 2-fold more IL-4 and IL-17A, respectively, but only slightly more IFNγ. The ability to provide a TcR-like signal capable of promoting T helper cell differentiation and cytokine synthesis was not common to all GPI-APs since cross-linking of Ly6A/E with mitogenic mAb did not promote substantial production of IFNγ, IL-4 or IL-17, although there was a substantial proliferative response. The preferential induction of RORγt and Th17 cytokine synthesis as a consequence of Thy-1 signaling suggests a default T helper cell response that may enhance host defense against extracellular pathogens.

## Introduction

Thy-1is a 25 kDa glycosylphosphatidylinositol-anchored protein (GPI-AP) that is highly expressed on the surface of mouse thymocytes and peripheral T cells (Pont, 1987; Haeryfar and Hoskin, 2004). As with several other T cell-associated GPI-APs (Malek et al., 1994), cross-linking Thy-1 molecules with mitogenic anti-Thy-1 monoclonal antibody (mAb; clone G7) in the presence of costimulatory syngeneic bone marrow-derived dendritic cells (BMDCs) results in T cell proliferation, interleukin (IL)-2 production and IL-2 receptor (CD25) expression (Haeryfar et al., 2003). The physiological ligand for T cell-associated Thy-1 has not yet been identified, although within the neurological compartment Thy-1 interacts with β_3_ integrin on astrocytes to promote astrocyte adhesion (Leyton et al., 2001), while leukocyte-associated α_m_β_2_ integrin promotes leukocyte adhesion to Thy-1-expressing endothelium (Wetzel et al., 2004). In addition, galactin-1, a soluble sugar binding protein, binds Thy-1 in a carbohydrate-dependent manner (Symons et al., 2000).

Since Thy-1 and other GPI-APs are localized within T cell lipid rafts, it is proposed that these GPI-APs induce T cell activation by the common mechanism of lipid raft aggregation and the subsequent activation of lipid raft-associated signaling molecules (Ilangumaran et al., 2000). Although T cell activation via Thy-1 crosslinking is at least partially dependent on expression of the complete T cell receptor (TcR) complex (Gunter et al., 1987), there are notable differences between Thy-1 and TcR stimulation (Furlong et al., 2017). Nevertheless, many of the same signaling molecules that are involved in TcR signaling have also been implicated in Thy-1 signaling. For example, Thy-1 signaling in T cells involves Ca^+2^ flux, activation of Lck, Fyn, and Zap-70 protein tyrosine kinases, mitogen-activated protein kinases (MAPKs), phospholipase C γ, protein kinase C, and phosphatidylinositol-3 kinase (Leyton et al., 1999; Haeryfar and Hoskin, 2001; Conrad et al., 2009; Furlong et al., 2017). Consistent with Thy-1 providing T cells with a TcR-like signal, Thy-1 stimulation in the presence of costimulatory BMDCs results in the development of fully armed cytotoxic T lymphocytes; however, Thy-1-induced cytotoxic T lymphocytes are deficient in granule-dependent cytotoxicity and function via the Fas/Fas ligand death receptor pathway (Kojima et al., 2000; Haeryfar et al., 2003; Furlong et al., 2017). Interestingly, Thy-1 crosslinking in the absence of costimulatory signaling causes CD4^+^ T cells to express CD25 and exhibit regulatory function without expression of the T regulatory cell lineage-specific transcription factor FoxP3 (Conrad et al., 2012). However, the precise function of Thy-1 in T cell activation, differentiation and effector function is far from being completely understood.

Th1, Th2, and Th17 cells are CD4^+^ T helper cell subsets with distinct cytokine profiles and biological functions (Fietta and Delsante, 2009). In the presence of interferon (IFN) γ and IL-12, naïve CD4^+^ T cells differentiate into Th1 cells following antigen recognition (Lighvani et al., 2001; Mullen et al., 2001). Environments rich in IL-4 and IL-2 favor Th2 differentiation (Swain et al., 1990), whereas the development of Th17 cells is promoted by transforming growth factor (TGF)-β, IL-1β, IL-6 and/or IL-21 (Veldhoen et al., 2006; Acosta-Rodriguez et al., 2007; Korn et al., 2007). Development of each T helper cell subset is reciprocally regulated, ensuring efficient activation of only the most appropriate effector mechanisms (Fietta and Delsante, 2009). Th1 cells produce cytokines such as IFNγ that support cell-mediated immune responses against malignant cells, viruses and other intracellular pathogens, whereas Th2 cells are a source of IL-4 and other cytokines that support humoral immunity and allow for the elimination of extracellular pathogens, including helminthes. On the other hand, IL-17 and additional proinflammatory cytokines synthesized by Th17 cells are critical for protection against Gram-negative bacteria, fungi, and certain protozoal pathogens. The production of signature cytokines by Th1, Th2, and Th17 cells is regulated by lineage-specific transcription factors: T-bet for Th1 cytokines, GATA3 for Th2 cytokines, and RORγt for Th17 cytokines (Li et al., 2014). Dysregulation of T helper cell subsets can result in the pathogenesis of immune-mediated inflammatory diseases (Hirahara and Nakayama, 2016). A more thorough understanding of how T helper cell subsets develop will suggest new strategies for the treatment of infectious diseases, autoimmune and allergic disorders, and cancer.

In this study, we explored the effect of Thy-1 signaling, in the context of costimulatory signals from BMDCs, on T cell synthesis of cytokines that are typically associated with Th1 (IFNγ), Th2 (IL-4), and Th17 (IL-17A) CD4^+^ T cell responses. TcR-activated T cells were used for comparison. We also compared expression of the lineage-specific transcription factors T-bet, GATA3 and RORγt by Thy-1- and TcR-activated T cells. In addition, the capacity of Thy-1 stimulation, under different T helper cell polarizing conditions, to induce the differentiation of CD4^+^ T cells into Th1, Th2, and Th17 T helper cell subsets was also determined and compared to the effect of TcR stimulation under the same polarizing conditions. We show for the first time that T cell stimulation via Thy-1 in the absence of a polarizing environment preferentially induced a Th17 response. Under polarizing conditions, Thy-1 signaling, like TcR stimulation, promoted CD4^+^ T cell differentiation into Th1, Th2, and Th17 subsets; however, Th1 responses were nearly equivalent whereas Th2 and Th17 responses were stronger in comparison to TcR-activated CD4^+^ T cells.

## Materials and Methods

### Animals

Adult female C57BL/6 mice, purchased at 6 to 8-weeks-of-age from Charles River Canada (Lasalle, QC, Canada), were housed in the Carleton Animal Care Facility at Dalhousie University and maintained on standard rodent chow and water supplied *ad libitium*. Animal protocols were approved by the Dalhousie University Committee on Laboratory Animals and were consistent with the Canadian Council on Animal Care Guidelines.

### Medium

RPMI 1640 medium (Sigma-Aldrich, Oakville, ON, Canada) was supplemented with 5% heat-inactivated fetal calf serum (FCS), 100 U/ml penicillin, 100 μg/ml streptomycin, 2 mM L-glutamate, and 5 mM 4-(2-hydroxyethyl)-1-piperazineethanesulfonic acid (HEPES) buffer (pH 7.4; Invitrogen; Burlington, ON, Canada). BMDC medium consisted of RPMI 1640 medium supplemented with 10% heat-inactivated FCS, 2 mM L-glutamine, 200 U/ml penicillin, 200 μg/ml streptomycin, 5 mM HEPES buffer and 50 μM β-mercaptoethanol (Sigma-Aldrich).

### Cytokines and Antibodies

Recombinant mouse IFNγ, IL-12, IL-4, IL-6, and recombinant human TGF-β1 were purchased from Peprotech (Rocky Hill, NJ, United States). Recombinant mouse GM-CSF was from R&D Systems, Inc. (Minneapolis, MN, United States). Anti-Thy-1 mAb (clone G7, rat IgG2c), fluorescein isothiocyanate (FITC)-conjugated anti-Thy-1.2 mAb (clone 30-H12, rat IgG2b; clone 53-2.1, rat IgG2a) and rat IgG2c were purchased from BD Biosciences (Mississauga, ON, Canada). Anti-TcRβ mAb (clone H57-597, hamster IgG), anti-Ly6A/E mAb (clone D7, ratIgG2a), anti-IL-4 mAb (clone 11B11, rat IgG1), anti-IFNγ mAb (clone R4-6A2, rat IgG1), anti-IL-12/IL-23 p40 (clone C17.8, rat IgG2a), anti-RORγt mAb (clone B2D, rat IgG1κ), hamster IgG, phycoerythrin (PE)-conjugated anti-GATA3 (clone TWAJ, rat IgG2b), PE-conjugated anti-T-bet mAb (clone 4B10, mouse IgG1), FITC-conjugated anti-CD62L mAb (clone MEL-14, rat IgG2a), PE-conjugated antiCD44 mAb (clone IM7, rat IgG2b), FITC-conjugated rat IgG2b, FITC-conjugated rat IgG2a, FITC-conjugated hamster IgG, PE-conjugated rat IgG2b, PE-conjugated mouse IgG1, and PE-conjugated hamster IgG were purchased from eBioscience, Inc. (San Diego, CA, United States). FITC-conjugated anti-CD3ε mAb (clone 145-2C11, hamster IgG), and PE-conjugated anti-TcRαβ mAb (clone H57-597, hamster IgG) were purchased from Cedarlane Laboratories, Inc. (Hornby, ON, Canada). Anti-actin antibody (clone I-19), horse radish peroxidase (HRP)-conjugated anti-goat IgG, and HRP-conjugated anti-rat IgG were from Santa Cruz Biotechnology (Santa Cruz, CA, United States).

### BMDC Preparation

BMDCs were prepared as described (Lutz et al., 1999). Briefly, tibias and femurs from euthanized mice were flushed with phosphate buffered saline (PBS) to create a single cell suspension. Erythrocytes were depleted by hypo-osmotic shock and the remaining bone marrow cells were resuspended in BMDC medium containing 20 ng/ml GM-CSF prior to being seeded into 6-well plates at 1 × 10^6^ cells/well. After culture for 7 days at 37°C in a humidified 5% CO_2_ incubator, non-adherent cells were treated with 1 μg/ml lipopolysaccharide (LPS, Sigma-Aldrich) for 24 h to promote dendritic cell maturation.

### T Cell Isolation

Spleen cell or lymph node cell suspensions were prepared in ice-cold PBS using a tissue homogenizer. Erythrocytes were depleted by hypo-osmotic shock. Highly purified (>98%) CD3^+^ T cells were obtained using the Pan T Cell Isolation MACS^®^ kit from Miltenyi Biotec (Cambridge, MA, United States), as per the manufacturer’s instructions. Highly purified CD4^+^ or CD8^+^ T cells were isolated by negative selection from lymph node cell preparations using CD4^+^ or CD8^+^ T Cell MACS^®^ isolation kits, as per the manufacturer’s instructions.

### T Cell Activation

T cells in fully supplemented RPMI 1640 medium were plated in either 96-well U-bottom (2.5 × 10^5^ cells/well) or 24-well flat-bottom plates (1.25–2.5 × 10^6^ cells/well) and activated with anti-TcRβ mAb, anti-Thy-1 mAb, anti-Ly6A/E in the presence of LPS-matured syngeneic BMDCs (8 × 10^3^ cells/well or 4–8 × 10^4^ cells/well, respectively) or 5 ng/ml phorbol 12-myristate 13-acetate (PMA), as indicated.

### CD4^+^ T Cell Polarization

CD4^+^ T cells were seeded into 24-well flat-bottom plates (1.25 × 10^6^ cells/well) and activated with LPS-matured syngeneic BMDCs (40 × 10^3^ cells/well) and anti-TcRβ mAb or anti-Thy-1 mAb under different Th cell polarizing conditions: Th1 – 5 ng/ml IL-12 and 10 μg/ml anti-IL-4 mAb; Th2 – 10 ng/ml IL-4, 10 μg/ml anti-IL-12 mAb and 10 μg/ml anti-IFNγ mAb; Th17 – 100 ng/ml IL-6, 1 ng/ml TGFβ1, 10 μg/ml anti-IFNγ mAb, and 10 μg/ml anti-IL-4 mAb (Stritesky et al., 2008). On day 6, T cells were harvested and were restimulated with 5 ng/ml PMA plus 500 ng/ml ionomycin.

### Tritiated-Thymidine Incorporation

Cultures performed in 96-well U-bottom plates were pulsed with 0.25 μCi of methyl ^3^H-thymidine ([^3^H]TdR; MP Biomedicals, Irvine, CA, United States) for 6 h. DNA was then harvested onto glass fiber filter mats using a Titer-Tek cell harvester (Skatron Instruments, Lier, Norway). [^3^H]TdR incorporation, which is a measure of DNA synthesis, was determined using a Beckman LS6000IC liquid scintillation counter (Beckman Coulter, Inc., Brea, CA, United States).

### Flow Cytometry

For detection of cell-surface molecules, T cells were stained with FITC- or PE-conjugated mAbs or the appropriate isotype control, both at 10 μg/ml concentration. Briefly, T cells were resuspended in flow cytometry buffer (1% bovine serum albumin [w/v] and 0.2% sodium azide [w/v] in PBS), the desired mAb was added, and cells were incubated on ice in the dark for 45 min. Cells were then washed and fixed with 1% paraformaldehyde in PBS prior to flow cytometric analysis.

For intracellular staining of T-bet and GATA3, T cells were permeabilized and fixed using the FoxP3 staining kit (eBioscience), according to the manufacturer’s instructions, and then stained with PE-conjugated anti-T-bet mAb (0.5 μg/ml), PE-conjugated anti-GATA3 mAb (0.06 μg/ml) or the appropriate isotype control for 45 min on ice in the dark. Cells were then washed and analyzed by flow cytometry.

For measurement of cell proliferation, T cells were labeled with 2.5 μM Oregon Green 488 dye (Invitrogen) in warm PBS for 10 min. Excess dye was inactivated by incubation for 30 min at 37°C in RPMI 1640 medium containing FCS. Cells were then washed and seeded at 2.5 × 10^6^ cells/well into 24-well plates for activation as previously described. At the end of culture, cells were harvested and serial halving of fluorescence, which represents a round of cell division, was detected by flow cytometry.

Fluorescence intensity of individual cells was determined using a FACSCaliber flow cytometer with CellQuest software (version 3.3; Becton Dickinson; Mississauga, ON, United States). Data were analyzed using FCS Express software (verson 3.0; De Novo Software; Thornhill, ON, United States).

### mRNA Expression

T cells were lysed in TRIzol reagent (Invitrogen) and total RNA was extracted as per the manufacturer’s instructions. RNA was quantified using a spectrophotometer and RNA purity was determined based on the A260/A280 ratio. Moloney murine leukemia virus reverse transcriptase (Invitrogen) was used to reverse transcribe RNA, following the manufacturer’s instructions. The resulting cDNA was then stored at -80°C for future use. Real time-polymerase chain reaction (RT-PCR) was carried out using the Quantifast SYBR-green RT-PCR kit (Qiagen; Mississauga, ON, United States). cDNA was amplified using the following primers: IFNγ, (F) 5′-ATG AAC GCT ACA CAC TGC ATC-3′, (R) 5′-CCA TCC TTT TGC CAG TTC CTC-3′; IL-4, (F) 5′-ACT TGA TGA GAG AGA TCA TCG GCA-3′, (R) 5′-AGC TCC ATG AGA ACA CTA GAG TT-3′; IL-17A, (F) 5′-CTC CAG AAG GCC CTC AGA CTA C-3′, (R) 5′-AGC TTT CCC TCC GCA TTA CAC AG-3′; and RNA polymerase II, (F) 5′-GCG GAT GAG GAT ATG CAA TAT GA-3′, (R) 5′-ACC AAG CCT TTC TCG TCA AAA TA-3′. RT-PCR reactions were performed using a MX3000P quantitative PCR machine (Stratagene; La Jolla, CA, United States). Cycling conditions were: 10 min activation step at 95°C, 40 amplification cycles at 95°C for 10 s, and 60°C for 30 s. Data were analyzed using Stratagene MxPro software, version 3.0. The size and integrity of RT-PCR products were verified using melt curve analysis and by running products on 3% agarose gels. Relative concentrations of mRNA were determined using the standard curve method, whereby standard curves are generated using serial dilutions of the cDNA from activated T cells. Cytokine mRNA levels were normalized to RNA polymerase II mRNA levels.

### Enzyme-Linked Immunosorbant Assay (ELISA)

Cell-free supernatants from T cell cultures were assayed for IFNγ, IL-4 and IL-17A content using ELISA kits from eBioscience or BD Biosciences, according to the manufacturer’s instructions. Absorbance at 450 nm with a wavelength correction for 570 nm was determined using an ELx800 UV universal microplate reader (Biotek Instruments, Inc., Winooski, VT, United States) and KCjunior software (version 1.17; Biotek Instruments, Inc.). SOFTmax^®^ PRO software (version 4.3; Molecule Devices, Corp., Sunnyvale, CA, United States) was used to determine cytokine concentrations from the absorbance readings.

### Western Blotting

Cells were lysed in ice-cold RIPA buffer (50 mM Tris-HCl, pH 7.5, 150 mM NaCl, 50 mM Na_2_HPO_4_, 0.25% sodium deoxycholate [w/v], 0.1% Nonidet P-40 [v/v], 5 mM ethylenediaminetetraacetic acid, and 5 mM ethyleneglycoltetraacetic acid) supplemented with fresh protease and phosphatase inhibitors (1 mM Na_3_VO_4_, 1 mM NaF, 1 mM phenylmethylsulfonyl fluoride, 1 μg/ml aprotinin, 1 μg/ml leupeptin, and 1 μg/ml pepstatin) for 15–30 min on ice. Cellular debris was removed from the lysates by centrifugation at 10,000 *g*. Total protein concentration was determined by Bradford assay (Bio-Rad, Hercules, CA, United States). Equal amounts of protein were added to sample buffer (200 mM Tris-HCl [pH 6.8], 30% glycerol [v/v], 6% sodium dodecyl sulfate [w/v], 15% β-mercaptoethanol [v/v], and 0.001% bromophenol [w/v]), which was then heated to 90–100°C for 5 min to promote protein denaturation. Lysates were stored at -80°C until use. Protein samples (10–20 μg protein/well) were loaded onto Tris-HCl acrylamide resolving gels and sodium dodecyl sulfate-polyacrylamide gel electrophoresis was used to separate proteins, which were then transferred onto nitrocellulose membranes using an iBlot^®^ Dry Blotting System (Invitrogen). Membranes were washed with Tris-buffered saline (TBS)-Tween-20 (TBST; 20 mM Tris-HCl [pH 7.6], 200 mM NaCl, 0.05% Tween-20 [v/v]) and blocked in TBST containing 5% fat-free milk powder [w/v] for 1 h at room temperature or overnight at 4°C with gentle rocking. Membranes were washed and then exposed to the primary antibody (1:200–1:1000 in TBST blocking solution) for 1 h at room temperature or overnight at 4°C with gentle rocking. Membranes were washed and then exposed to the appropriate HRP-conjugated secondary antibody (1:1000 in TBST blocking solution) for 1 h at room temperature with gentle rocking. Membranes were washed and reacted with enhanced chemiluminescence reagents (GE Healthcare, Baie d’Urfe, Quebec, CA, United States) for 1 min. Protein bands were visualized by exposure to X-ray film, which was developed in a Kodak X-OMAT 1000A automated X-ray developer.

### Statistical Analysis

Data were analyzed using Instat software (GraphPad Software, Inc., San Diego, CA, United States). Student’s *t*-test or one-way analysis of variance (ANOVA) with the Bonferroni multiple comparisons post-test were used as appropriate.

## Results

### CD4^+^ and CD8^+^ T Cell Expression of, and Activation via, Thy-1

**FIGURE 1:**
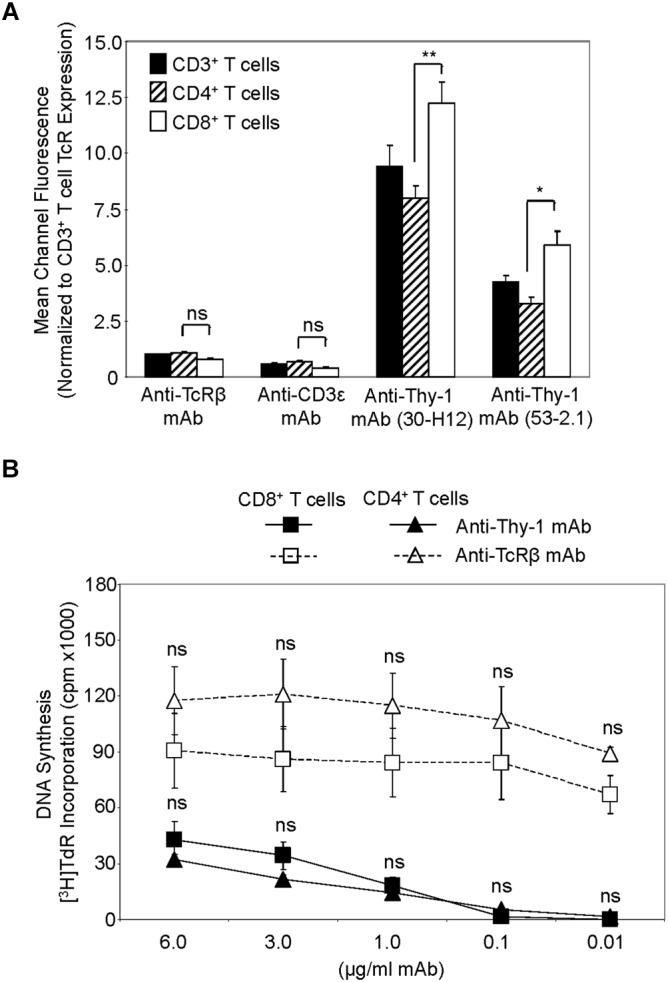
Thy-1 versus TcR expression by CD4^+^ and CD8^+^ T cells, and relative response to Thy-1 and TcR signaling. **(A)** Highly purified CD3^+^ T cells, CD4^+^ T cells and CD8^+^ T cells were stained with anti-TcRαβ-PE, anti-CD3ε-FITC, anti-Thy-1-FITC (clone 30-H12), anti-Thy-1-FITC (clone 53-2.1), or the appropriate FITC- or PE-labeled isotype control and analyzed by flow cytometry. Data are expressed as the average mean channel fluorescence normalized to TcR expression on CD3^+^ T cells. Data are the mean ± SEM of three independent experiments; ^∗^*p* < 0.05; ^∗∗^*p* < 0.001; and ns, not-significant, as determined by ANOVA and the Bonferroni multiple comparisons post-test. **(B)** CD4^+^ T cells or CD8^+^ T cells with or without LPS-matured BMDCs, were seeded in triplicate into 96-well round-bottom plates, and then cultured in the presence of the indicated concentrations of anti-Thy-1 mAb (clone G7), anti-TcRβ mAb or isotype control for 72 h. Wells were pulsed with [^3^H]TdR 6 h before the end of culture at which time the cells were harvested and DNA synthesis was determined based on [^3^H]TdR incorporation. Background proliferation was controlled for by subtraction of experimental cpm from cpm of T cells and BMDC cultured alone (7288 ± 1488 for CD8^+^ T cells and BMDCs, and 44157 ± 11919 for CD4^+^ T cells and BMDCs) and are the mean ± SEM of three independent experiments; ns, not significant, as determined by ANOVA and the Bonferroni multiple comparisons post-test when the proliferation of CD4^+^ T cells was compared to that of CD8^+^ T cells that were activated by anti-Thy-1 or anti-TcRβ mAb.

We first compared Thy-1 and TcR expression by CD4^+^ and CD8^+^ T cells, and their responsiveness to stimulation with anti-Thy-1 mAb versus anti-TcRβ mAb, in the presence of syngeneic BMDCs to provide costimulation. Flow cytometric analysis of highly purified CD4^+^ and CD8^+^ T cells, as well as unfractionated CD3^+^ T cells, labeled with two different fluorescent anti-Thy-1 mAbs showed a significant difference in mean channel fluorescence, indicating that Thy-1 was more abundant on CD8^+^ T cells than on CD4^+^ T cells; in contrast, CD8^+^ and CD4^+^ T cell expression of TcRβ and CD3ε was similar (Figure 1A). Surprisingly, stimulation of highly purified CD4^+^ and CD8^+^ T cells with anti-Thy-1 mAb in the presence of BMDCs resulted in similar levels of DNA synthesis (Figure 1B), suggesting that differences in Thy-1 expression may not be functionally significant. DNA synthesis by CD4^+^ and CD8^+^ T cells in response to stimulation with anti-TcRβ mAb in the presence of BMDCs was substantially greater than the proliferative response to stimulation with anti-Thy-1 mAb under the same conditions, and at all mAb concentrations tested, which was in line with our earlier finding that Thy-1 cross-linking provides a weaker activating signal than TcR cross-linking (Furlong et al., 2017). Subsequent experiments used anti-Thy-1 mAb and anti-TcRβ mAb at 6 μg/ml since this concentration of mAb induced a level of T cell activation that was not statistically different from that obtained with half the amount of mAb.

### Differential Cytokine Response of Thy-1-Stimulated T Cells

**FIGURE 2:**
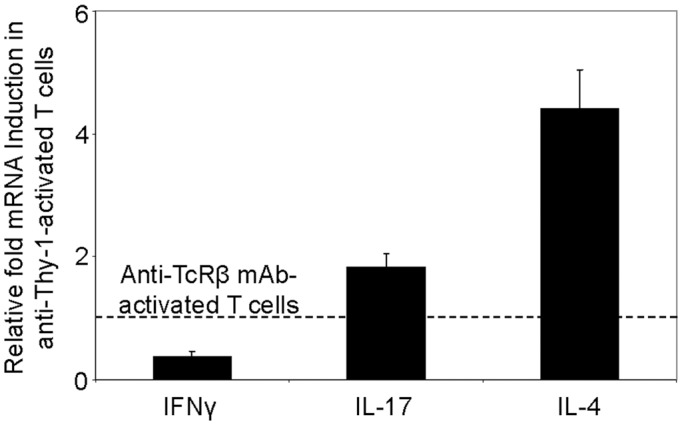
Differential induction of T helper subset-associated cytokine mRNA by Thy-1 and TcR stimulation. Highly purified CD3^+^ T cells with or without LPS-matured BMDCs were seeded into 24-well plates and then cultured in the presence or absence of 6 μg/ml anti-Thy-1 mAb (clone G7), anti-TcRβ mAb or appropriate isotype control for 24 h. Total RNA was isolated and used to generate cDNA. RT-PCR with primers specific for IFNγ, IL-17, IL-4 mRNA was performed. Pol II expression was used as a loading control. Relative expression of each cytokine mRNA was calculated using the standard curve method and normalized to the TcR-activated T cells. Data are the mean ± SEM of at least three separate experiments.

**FIGURE 3:**
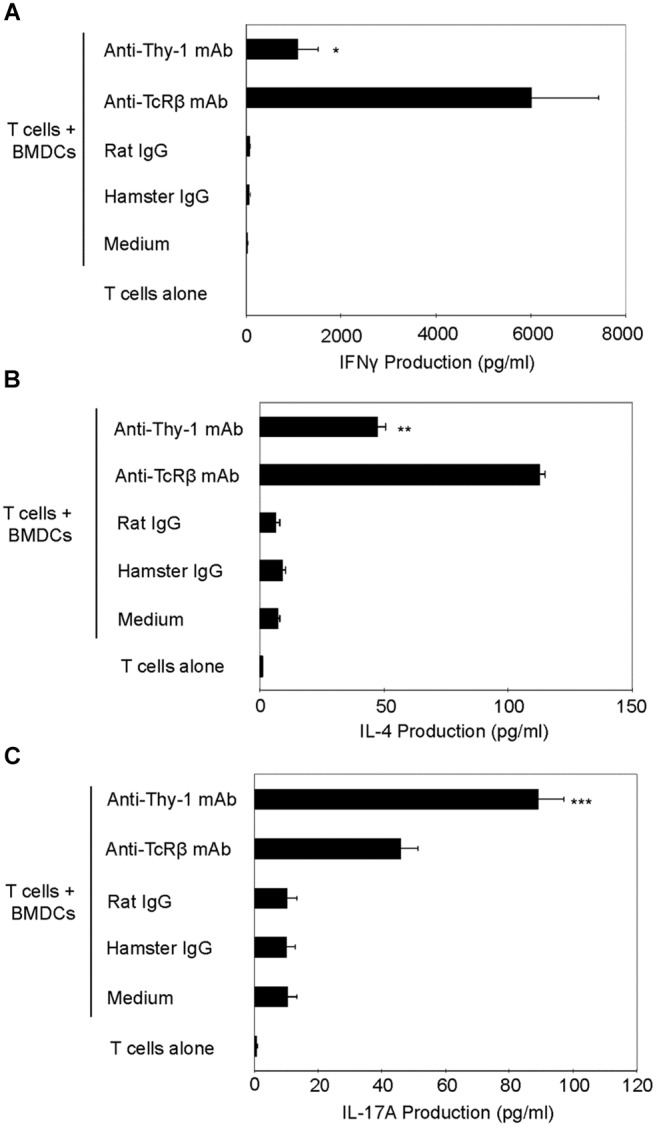
Thy-1 signaling induces more IL-17A but less IL-4 and IFNγ synthesis by CD3^+^ T cells in comparison to TcR signaling. **(A–C)** Highly purified CD3^+^ T cells with or without LPS-matured BMDCs were seeded in quadruplicate into 96-well round-bottom plates and then cultured in the presence of 6 μg/ml anti-Thy-1 mAb (clone G7), anti-TcRβ mAb or the appropriate isotype control for the 24 h. Supernatants were isolated and analyzed by ELISA for **(A)** IFNγ **(B)** IL-4, and **(C)** IL-17A. Data shown are the mean of at least three separate experiments ± SEM; ^∗^*p* < 0.05; ^∗∗^*p* < 0.01; ^∗∗∗^*p* < 0.001; and ns, not significant, when compared to T cells activated with anti-TcRβ mAb and LPS-matured BMDCs, as determined by the Bonferroni multiple comparisons post-test.

We next used RT-PCR to compare the effect of Thy-1 and TcR stimulation of CD3^+^ T cells on cytokine mRNA expression associated with Th1 (IFNγ), Th2 (IL-4), and Th17 (IL-17) cells. Flow cytometric analysis revealed that 58% of CD3^+^ T cells were CD44^low-medium^CD62L^+^ (naïve phenotype) and 15% were CD44^high^CD62L^+^ (effector/memory phenotype). Figure 2 shows that, in comparison to TcR-activated T cells, Thy-1-activated T cells expressed substantially less IFNγ mRNA at 24 h post-activation; in contrast, IL-4 and IL-17A mRNA expression by Thy-1-activated T cells was significantly greater than that of TcR-activated T cells. ELISA measurements showed that at 24 h post-activation, Thy-1-stimulated CD3^+^ T cell cultures contained significantly less IFNγ (Figure 3A) and more IL-17A (Figure 3C) than TcR-stimulated CD3^+^ T cell cultures. In contrast, high levels of IL-4 mRNA expressed by Thy-1 stimulated T cells relative to TcR-stimulated T cells did not correlate with IL-4 protein expression, which was greater in TcR-stimulated T cells relative to Thy-1-stimulated T cells (Figure 3B).

### Differential Expression of the T Helper Cell Lineage-Specific Transcription Factors by Thy-1-Stimulated T Cells

**FIGURE 4:**
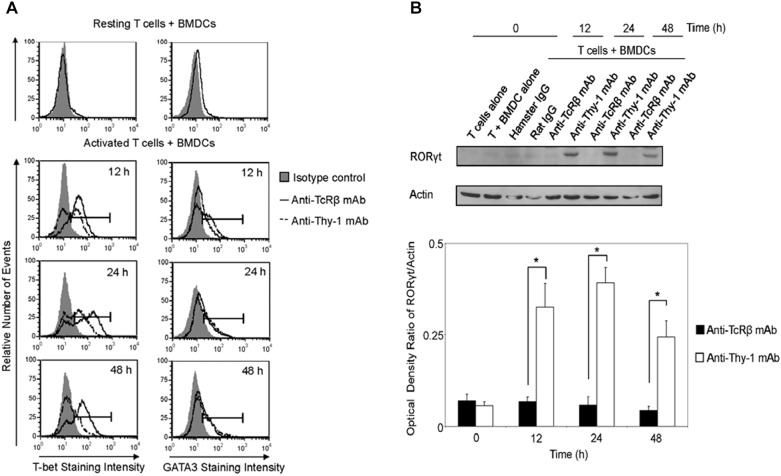
Thy-1 signaling induces lower T-bet expression and higher GATA3 and RORγt expression in CD3^+^ T cells in comparison to TcR signaling. **(A)** Highly purified CD3^+^ T cells were seeded in quadruplicate into 96-well round-bottom plates with or without LPS-matured BMDCs, and were then cultured in the presence of 6 μg/ml anti-Thy-1 mAb (clone G7), anti-TcRβ mAb, or the appropriate isotype control for the indicated times. Cells were then fixed, permeabilized, and stained with PE-labeled anti-T-bet mAb, PE-labeled anti-GATA3 mAb, or the appropriate PE-labeled isotype control. Expression of T-bet and GATA3 was measured by flow cytometry. Data are representative of three separate experiments. **(B)** Highly purified CD3^+^ T cells with or without LPS-matured BMDCs were seeded into 24-well plates and then cultured in the absence or presence of 6 μg/ml anti-Thy-1 mAb (clone G7), anti-TcRβ mAb, or the appropriate isotype control for the indicated times. Cell lysates were prepared and RORγt protein (58 kDa) levels were assessed by western blotting. Blots were reprobed for β-actin (42 kDa) to confirm equal loading of protein. Data are representative of three independent experiments. Optical density ratios were calculated by comparing the density of individual RORγt bands from three independent experiments with the corresponding β-actin bands. Data are shown as mean ± SEM; ^∗^*p* < 0.001, as determined by the Student’s *t*-test.

We next determined whether differential cytokine synthesis by CD3^+^ T cells in response to Thy1- and TcR-signaling was associated with differential expression of the lineage-specific transcription factors, T-bet (Th1), GATA3 (Th2), and RORγt (Th17). As shown in Figure 4A, flow cytometry revealed that T-bet expression increased at 12, 24, and 48 h post-stimulation of CD3^+^ T cells with anti-Thy-1 or anti-TcRβ mAb; however, Thy-1-activated T cells expressed less T-bet than TcR-activated T cells. A similar increase in GATA-3 expression by CD3^+^ T cells at 24 and 48 h was observed following Thy-1- and TcR-stimulation. Although not sustained, more GATA-3 was expressed by Thy-1-activated CD3^+^ T cells in comparison to TcR-activated CD3^+^ T cells at 12 h post-stimulation. Co-cultures of unstimulated CD3^+^ T cells and BMDCs expressed neither T-bet nor GATA3. Western blot analysis (Figure 4B) showed that Thy-1-activated CD3^+^ T cells exhibited a marked increase in RORγt expression at 12, 24, and 48 h post-stimulation, whereas TcR-activated CD3^+^ T cells failed to up-regulate RORγt expression. Co-cultures of unstimulated CD3^+^ T cells and BMDCs failed to express RORγt.

### Thy-1 Signaling Promotes CD4^+^ T Cell Differentiation Into T Helper Cell Subsets

**FIGURE 5:**
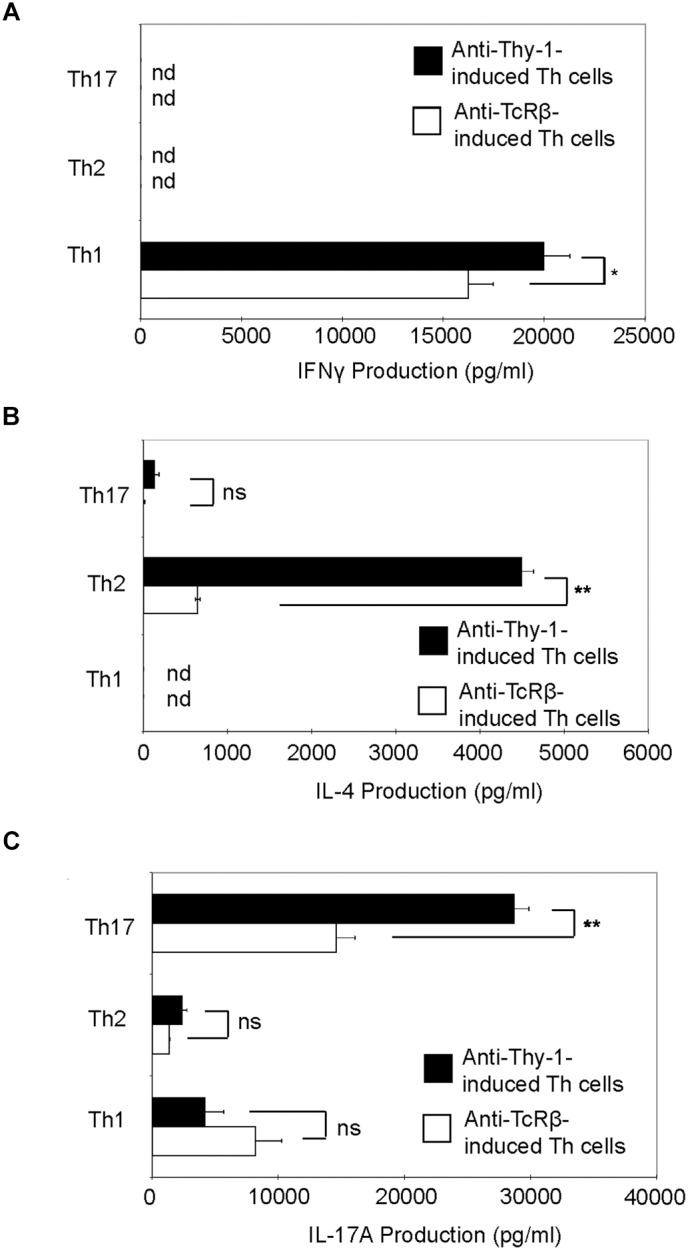
Thy-1 signaling promotes Th1, Th2, and Th17 effector cell development in the presence of T helper cell subset-polarizing conditions. Highly purified CD4^+^ T cells with or without LPS-matured BMDCs were seeded into 24-well flat-bottom plates and then stimulated with 6 μg/ml anti-Thy-1 mAb (clone G7) or anti-TcRβ mAb under Th1 (IL-12, anti-IL-4 mAb), Th2 (IL-4, anti-IL-12 mAb, anti-IFNγ mAb) or Th17 (IL-6, TGF-β, anti-IFNγ mAb, and anti-IL-4 mAb) polarizing conditions for 6 days. Polarized Th cells were washed, rested for 6 h and an equal number of viable cells were replated in 24-well flat-bottom plates, followed by restimulation with 5 ng/ml PMA and 500 ng/ml ionomycin and culture for 24 h. Supernatants were isolated and analyzed by ELISA for **(A)** IFNγ, **(B)** IL-4, and **(C)** IL-17A production. Data shown as mean ± SD are representative of three independent experiments; ^∗^*p* = 0.02; ^∗∗^*p* < 0.001; and ns, not significant, as determined by Student’s *t*-test.

Th1, Th2, and Th17 T cell development is governed by the cytokine environment in which antigen-dependent CD4^+^ T cell activation occurs (Fietta and Delsante, 2009). To determine whether Thy-1 stimulation supports Th1, Th2, and Th17 T cell differentiation, highly purified CD4^+^ T cells were activated with anti-Thy-1 mAb in the presence of BMDCs under Th1-polarizing (IL-12, anti-IL-4 blocking mAb), Th2-polarizing (IL-4, and blocking mAb against IFNγ, and IL-12) or Th17-polarizing (IL-6, TGF-β1 and blocking mAb against IL-4 and IFNγ) conditions for 6 days. Flow cytometric analysis revealed that 61% of CD4^+^ T cells were CD44^low-medium^CD62L^+^ (naïve phenotype) and 17% were CD44^high^CD62L^+^ (effector/memory phenotype). For comparison, parallel cultures were stimulated with anti-TcRβ mAb instead of anti-Thy-1 mAb. After polarization, CD4^+^ T cells were restimulated with PMA/ionomycin, and IFNγ, IL-4 and IL-17A content in culture supernatants was determined by ELISA. As shown in Figure 5, CD4^+^ T cells that were activated with anti-Thy-1 mAb (or anti-TcRβ mAb) under Th1-, Th2-, and Th17-polarizing conditions expressed the signature cytokines IFNγ (Figure 5A), IL-4 (Figure 5B), and IL-17A (Figure 5C), respectively. This finding is consistent with Thy-1 providing a TcR-like signal during T cell activation. Interestingly, in comparison to TcR signaling, Thy-1-stimulated CD4^+^ T cell cultures contained nearly sevenfold more IL-4 and twofold more IL-17A under Th2 and Th17 polarizing conditions, respectively, but only slightly more IFNγ when activated in a Th1 polarizing environment, indicating that Thy-1 signaling is a strong inducer of T helper cell subset differentiation under polarizing conditions, especially Th2 and Th17 responses.

### Differential Expression of Lineage-Specific Transcription Factors by Th1, Th2, and Th17 Cells Induced by Thy-1 Versus TcR Stimulation

**FIGURE 6:**
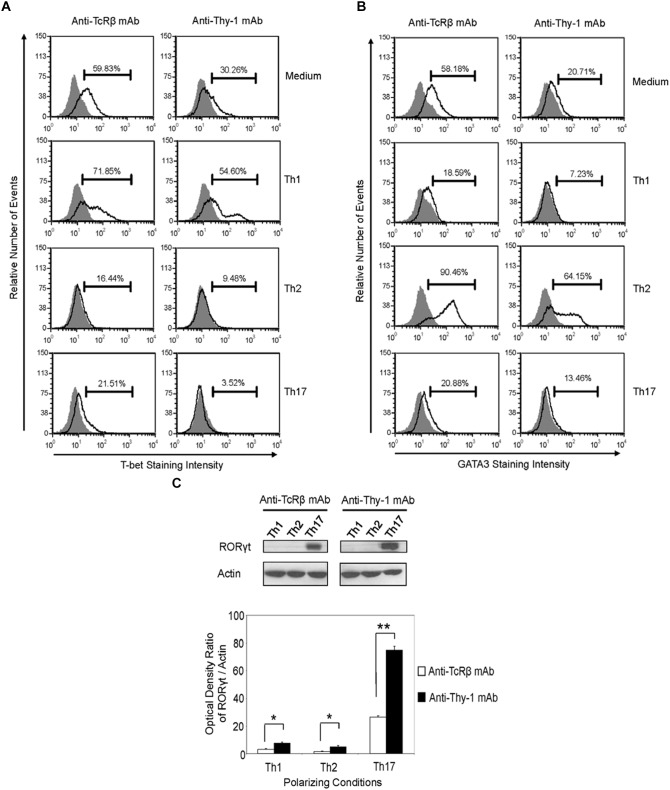
Thy-1-induced Th1 and Th2 cells express less T-bet and GATA3, respectively, whereas Thy-1-induced Th17 cells express more RORγt than TcR-induced counterparts. Highly purified CD4^+^ T cells with or without LPS-matured BMDCs were seeded into 24-well flat-bottom plates, and then stimulated with 6 μg/ml anti-Thy-1 mAb (clone G7) or anti-TcRβ mAb in the presence of medium alone or under Th1 (IL-12, anti-IL-4 mAb), Th2 (IL-4, anti-IL-12 mAb, anti-IFNγ mAb) or Th17 (IL-6, TGF-β, anti-IFNγ mAb, and anti-IL-4 mAb) polarizing conditions for 6 days. Cells were then fixed, permeabilized and stained for intracellular **(A)** T-bet (open peak) or **(B)** GATA3 (open peak), and compared with the appropriate isotype control (closed peak). Expression of T-bet and GATA3 was measured by flow cytometry. Data are representative of three separate experiments. **(C)** Cell lysates were prepared and RORγt protein (58 kDa) levels were assessed by western blotting. Blots were reprobed for β-actin (42 kDa) to confirm equal protein loading. Data are representative of three independent experiments. Optical density ratios were calculated by comparing the density of individual RORγt bands from three independent experiments with the corresponding β-actin bands. Data shown are the mean ± SEM; ^∗^*p* < 0.05; ^∗∗^*p* < 0.001, as determined by the Student’s *t*-test.

Differentiation of Thy-1-stimulated CD4^+^ T cells into Th1, Th2, and Th17 T cell subsets under the appropriate T helper cell polarizing conditions implied expression of the corresponding lineage-specific transcription factors. Indeed, highly purified CD4^+^ T cells that were activated with anti-Thy-1 mAb in the presence of BMDCs under Th1 polarizing conditions showed increased T-bet expression that was not evident when Th2 or Th17 polarizing conditions were used (Figure 6A). As expected, this was also the case with TcR-stimulated CD4^+^ T cells. In addition, both Thy-1- and TcR-stimulated CD4^+^ T cells expressed GATA3 when activated under Th2 polarizing conditions but not under Th1 or Th17 polarizing conditions (Figure 6B). In comparison to TcR-induced Th1 and Th2 cells, slightly less T-bet and GATA3 was expressed by Thy-1-induced Th1 and Th2 cells, respectively. In contrast, Th17 cells that were induced by Thy-1 signaling under Th17 polarizing conditions expressed significantly more RORγt than Th17 cells that developed in response to TcR stimulation (Figure 6C). Th1 and Th2 cells that were stimulated with anti-Thy-1 mAb also expressed RORγt, albeit at a much lower level than Th17 cells that arose because of TcR or Thy-1 stimulation under Th17 polarizing conditions.

### Ly6A/E-Stimulated T Cells Proliferate but Fail to Produce Th Cell Subset-Specific Cytokines

**FIGURE 7:**
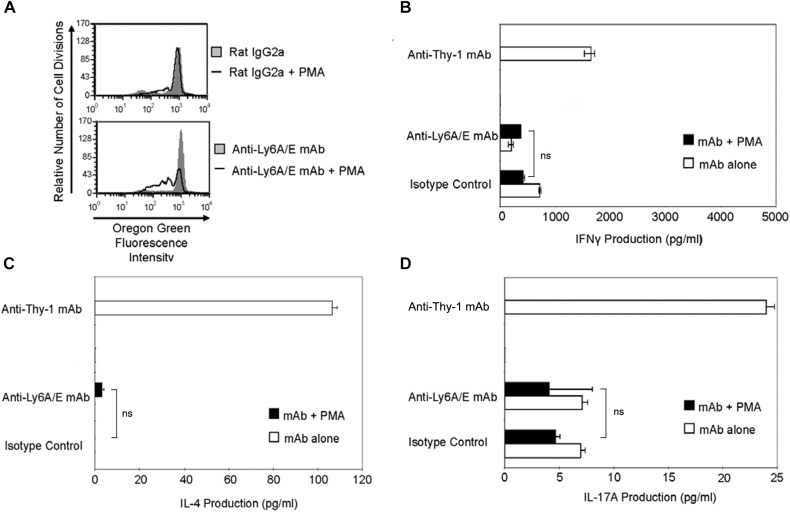
Ly6A/E stimulation of T cells induces a weak proliferative response but minimal IFNγ, IL-4, and IL-17A synthesis. **(A)** Highly purified CD3^+^ T cells were labeled with 2.5 μM Oregon Green 488 dye and seeded in duplicate into 96-well round-bottom plates. T cells were then cultured in the presence of 6 μg/ml anti-Ly6A/E mAb or the appropriate isotype control with or without 5 ng/ml PMA for the indicated times. Oregon Green 488 staining of pooled T cells was determined by flow cytometry. Fluorescence decreases with each round of cell division. Data shown are representative of three separate experiments. **(B)** CD3^+^ T cells with or without LPS-matured BMDCs were seeded into 96-well round-bottom plates and then cultured in the presence of 6 μg/ml anti-Thy-1 mAb (clone G7) or anti-Ly-6A/E mAb with or without 5 ng/ml PMA, respectively, or the appropriate isotype control for 24 h. Supernatants were isolated and analyzed by ELISA for **(B)** IFNγ, **(C)** IL-4, and **(D)** IL-17A content. Data shown as mean ± SD of three technical replicates are representative of two independent experiments; ns, not significant by Student’s *t*-test.

It is currently unclear whether the ability to provide a TcR-like signal that induces T helper cell subset cytokine synthesis is common to all T cell-associated GPI-APs or is unique to Thy-1. Ly6A/E is a GPI-AP that is known to promote T cell proliferation when cross-linked with mitogenic anti-Ly6A/E mAb in the presence of accessory cells and PMA (Malek et al., 1986). We therefore stimulated CD3^+^ T cells with mitogenic anti-Ly6A/E mAb without or with PMA and measured cell proliferation by Oregon Green 488 staining, as well as IFNγ, IL-4, and IL-17A levels in culture supernatants by ELISA. PMA was used instead of BMDCs because we were unable to detect T cell proliferation following stimulation with anti-Ly6A/E mAb in the presence of BMDCs (data not shown), suggesting a deficiency in 1,2-diacylglycerol-dependent signaling following Ly6A/E crosslinking. As expected, CD3^+^ T cells proliferated in the presence of anti-Ly6A/E mAb plus PMA (Figure 7A); however, in comparison to stimulation with anti-Thy-1 mAb in the presence of BMDCs, there was little or no production of IFNγ (Figure 7B), IL-4 (Figure 7C), and IL-17A (Figure 7D) when T cells were stimulated with anti-Ly6A/E mAb plus PMA. These data indicate that not all GPI-APs are able to signal for robust T helper cell cytokine synthesis.

## Discussion

The differentiation of CD4^+^ T cells into phenotypically distinct T helper cell subsets in response to antigenic stimulation is crucial for an immune response that is appropriately tailored for optimal host defense (Fietta and Delsante, 2009). In this study, we provide evidence for the first time that the TcR-like signal induced by Thy-1 crosslinking resulted in the development of functional T helper cell subsets. Moreover, in comparison to TcR signaling, Thy-1 signaling in the absence of polarizing conditions preferentially promoted the synthesis of IL-17A over IFNγ and IL-4, which suggests a fundamental difference between signaling pathways associated with Thy-1 and the TcR. It is important to note that cytokine mRNA levels did not exactly correlate with protein expression. The failure of a gene’s transcript level to predict its protein level is a well-known phenomenon that has been attributed to various factors, including translation efficiency and differences between mRNA and protein stability (Vogel et al., 2010; Schwanhäusser et al., 2011). Thy-1-stimulated T cells also showed lower expression of the Th1-defining transcription factor T-bet, which is expressed following TcR stimulation in the presence of IL-12 (Szabo et al., 2000). LPS-matured BMDCs secrete abundant IL-12 (Tada et al., 2004); however, in preliminary experiments we have observed that, in comparison to TcR-activated T cell cultures, significantly less IL-12 protein was present in Thy-1-stimulated T cell cultures (data not shown), which may account for reduced T-bet-dependent IFNγ expression. IL-4 synthesis by Thy-1-stimulated T cells was associated with an early and transient increase in expression of the Th2-defining transcription factor GATA3; at later time points Thy-1- and TcR-stimulated T cells expressed equivalent amounts of GATA3. Thy-1 crosslinking generates a weaker T cell-activating signal than crosslinking of TcRs (Furlong et al., 2017), which may account for the transient increase in GATA3 expression since a weak TcR signal favors the development of Th2 cells (Pfeiffer et al., 1995). Strikingly, Thy-1 signaling preferentially induced expression of the Th17-defining transcription factor RORγt, which regulates IL-17 synthesis by binding directly to the *IL-17* promoter (Zhang et al., 2008). It is known that a strong TcR signal favors IL-17 production and Th17 differentiation in mouse T cells (Gomez-Rodriguez et al., 2009); hence, the difference between Thy-1 and TcR signal strength was not consistent with preferential RORγt expression and IL-17A synthesis by Thy-1-stimulated T cells. Rather, there appears to be a fundamental difference between Thy-1- and TcR-associated signaling pathways that regulate Th17 development. Since additional transcription factors such as Runx1 and STAT3 collaborate with RORγt to promote optimal *IL-17* gene transcription (Wei et al., 2007; Zhang et al., 2008), it will be important in future studies to determine whether Thy-1 signaling uniquely upregulates and/or activates any additional Th17-related transcription factors relative to TcR signaling.

In contrast to T cell-BMDC co-cultures, Thy-1 stimulation of CD4^+^ T cells in the context of Th1, Th2, or Th17 polarizing cytokine environments clearly promoted Th1, Th2, and Th17 differentiation, respectively, indicating that Thy-1 provided an antigen-independent TcR-like signal that was sufficient to induce T helper cell subset differentiation. Moreover, Thy-1-activated CD4^+^ T cells showed greater production of signature cytokines upon restimulation than did TcR-stimulated CD4^+^ T cells, which suggests more efficient T helper cell polarization. Interestingly, IL-4 and IL-17A synthesis by Thy-1-stimulated CD4^+^ T cells was markedly increased in comparison to TcR-stimulated CD4^+^ T cells, suggesting that in the appropriate polarizing environment Thy-1 signaling preferentially promotes IL-4 and IL-17A synthesis. In contrast, IFNγ synthesis was only slightly increased following Thy-1 versus TcR stimulation of CD4^+^ T cells. Surprisingly, in comparison to TcR stimulation, Thy-1 stimulated CD4^+^ T cells expressed less T-bet and GATA3 under Th1 and Th2 polarizing conditions, respectively, suggesting that preferential activation of additional transcription factors involved in IFNγ and IL-4 gene expression (Kim et al., 1999; Samten et al., 2008) may contribute to increased synthesis of signature cytokines by Thy-1 stimulated CD4^+^ T cells. On the other hand, enhanced IL-17A synthesis was correlated with increased RORγt expression by Thy-1-stimulated CD4^+^ T cells.

The ability of Thy-1 to provide an antigen-independent TcR-like signal that promotes Th cell differentiation is not a common feature of all T cell-associated GPI-APs since mAb-mediated crosslinking of Ly6A/E in the presence of PMA failed to induce substantial IFNγ, IL-4, or IL-17A synthesis by T cells in comparison to Thy-1 stimulation. However, like Thy-1 signaling, Ly6A/E signaling resulted in T cell proliferation, suggesting at least some induction of IL-2 needed to support T cell replication. Although signaling via other T cell-associated GPI-APs such as Ly6A/E may not support T helper cell differentiation, these cell-surface molecules may still affect T helper cell differentiation. For example, cellular prion protein has been implicated in the optimal production of T cell cytokines since T cells from cellular prion protein-deficient mice generate less IFNγ, IL-4, and IL-17 in response to TcR-stimulation and have altered responses to infection and autoantigens (Ingram et al., 2009).

## Conclusion

**FIGURE 8:**
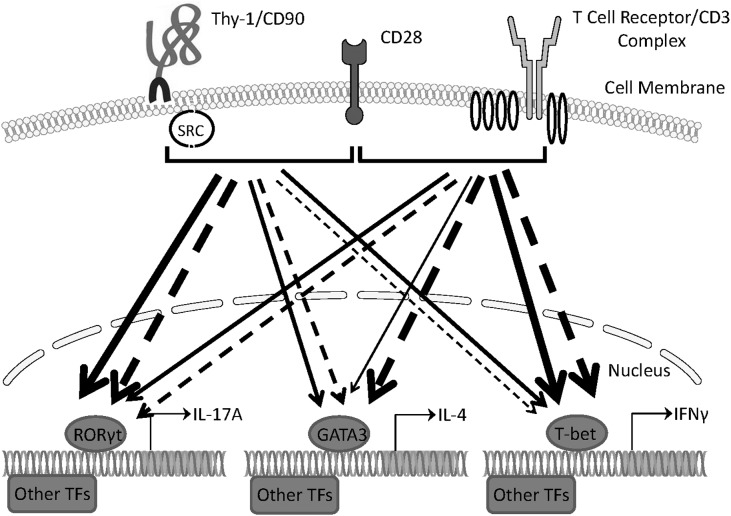
Schematic comparing Th1, Th2, and Th17 responses induced by Thy-1 and TcR signaling in the absence or presence of polarizing environments. In the presence of a costimulatory signal from CD28, Thy-1 signaling in the absence of a T helper cell subset polarizing environment (dashed lines) favors a relatively strong Th17 response whereas TcR signaling favors Th1 and Th2 responses. In a T helper cell subset polarizing environment (solid lines), Thy-1 signaling is more effective than TcR signaling at inducing both Th2 and Th17 responses but induced a similar Th1 response. Thickness of lines indicates relative strength of the signal from Thy-1 versus TcR. TF denotes transcription factor.

Our findings provide evidence that Thy-1 signaling in the context of costimulation provided by BMDCs is sufficient to promote the differentiation of T helper cell effector subsets, albeit with different outcomes depending on whether or not a polarizing environment is present. Figure 8 summarizes the effects of Thy-1 and TcR signaling in the absence or presence of Th1, Th2, or Th17 polarizing conditions. Our findings are consistent with a model in which Thy-1 crosslinking results in a weak TcR-like signal that preferentially promotes Th17 development in a non-polarizing environment, and Th2 and Th17 development under T helper cell subset polarizing conditions. We speculate that under physiological conditions, Thy-1 crosslinking by its physiological ligand, in combination with appropriate costimulatory signals, may result in enhanced antigen-independent host defense against extracellular pathogens.

## Author Contributions

SF and DH designed the study. SF and JG performed the experiments. SF, MC, JG, and DH analyzed the data and wrote the manuscript.

## Conflict of Interest Statement

The authors declare that the research was conducted in the absence of any commercial or financial relationships that could be construed as a potential conflict of interest.
